# Probiotic *Lactobacillus* rhamnosus GG mono-association suppresses human rotavirus-induced autophagy in the gnotobiotic piglet intestine

**DOI:** 10.1186/1757-4749-5-22

**Published:** 2013-08-07

**Authors:** Shaoping Wu, Lijuan Yuan, Yongguo Zhang, Fangning Liu, Guohua Li, Ke Wen, Jacob Kocher, Xingdong Yang, Jun Sun

**Affiliations:** 1 Department of Biochemistry, Rush University, Cohn Research Building, 1735 W. Harrison Street, Chicago, IL 60612, USA; 2 Department of Biomedical Sciences and Pathobiology, Virginia-Maryland Regional College of Veterinary Medicine, Virginia Polytechnic Institute and State University, Integrated Life Science Building, 1981 Kraft Dr, Blacksburg, VA 24061-0913, USA

**Keywords:** Autophagy, Apoptosis, Diarrhea, Gnotobiotic pig, *Lactobacillus rhamnosus* GG, Infectious disease, Intestinal inflammation, Intestinal injury, Probiotics, Rotavirus

## Abstract

**Background:**

Human rotavirus (HRV) is the most important cause of severe diarrhea in infants and young children. Probiotic *Lactobacillus rhamnosus* GG (LGG) reduces rotavirus infection and diarrhea. However, the molecular mechanisms of LGG-mediated protection from rotavirus infection are poorly understood. Autophagy plays an essential role in responses to microbial pathogens. However, the role of autophagy in HRV infection and LGG treatment is unknown. We hypothesize that rotavirus gastroenteritis activates autophagy and that LGG suppresses virus-induced autophagy and prevents intestinal damage in infected piglets.

**Methods:**

We used LGG feeding to combat viral gastroenteritis in the gnotobiotic pig model of virulent HRV infection.

**Results:**

We found that LGG feeding did not increase autophagy, whereas virus infection induced autophagy in the piglet intestine. Virus infection increased the protein levels of the autophagy markers ATG16L1 and Beclin-1 and the autophagy regulator mTOR. LGG treatment during viral gastroenteritis reduced autophagy marker expression to normal levels, induced apoptosis and partially prevented virus-induced tissue damage.

**Conclusion:**

Our study provides new insights into virus-induced autophagy and LGG suppression of uncontrolled autophagy and intestinal injury. A better understanding of the antiviral activity of LGG will lead to novel therapeutic strategies for infant infectious diseases.

## Introduction

Autophagy is a lysosome-mediated catabolic pathway that occurs ubiquitously in all eukaryotic cells [1]. It is an immune mechanism that removes intracellular pathogens by the degradation of dysfunctional intracellular organelles. Mammalian autophagy involves initiation, nucleation, elongation, closure, maturation, and degradation [2]. Impaired autophagy has been linked to the pathogenesis of various diseases, including inflammatory bowel disease (IBD), necrotizing enterocolitis (NEC), tuberculosis neurodegeneration, and aging. Polymorphisms in two autophagy-related genes—*ATG16L1* and interferon-regulated GTPase (*IRGM*)—are strongly correlated with Crohn’s disease (CD) [3,4]. Autophagy occurs in the intestinal epithelium of neonatal piglets [5], in the intestinal epithelium of NEC patients and in the ileum of NEC rats [6]. Multiple autophagy-related proteins control autophagosome formation. Activated mTOR, VPS34, and Beclin-1 (a mammalian homolog of yeast Atg6) are required to induce autophagy. Although it is unclear whether autophagy is a protective response or a deleterious process, it is clear that uncontrolled autophagy has adverse effects in certain pathological conditions, ultimately causing cell death.

Human rotavirus (HRV) is the most important cause of severe dehydrating diarrhea in infants and young children worldwide. Rotavirus hijacks autophagy and uses its membranes to transport viral proteins for the assembly of infectious particles at the sites of virus replication; inhibition of the autophagy signaling pathway blocks virus production [7]. *Lactobacillus rhamnosus* GG (LGG) is a probiotic strain that has reduced rotavirus diarrhea in many clinical trials [8-14]. The benefits of LGG therapy include reduced duration and incidence of rotavirus diarrhea, diminished rotavirus shedding, and increased production of rotavirus-specific antibodies. However, the molecular mechanisms of LGG protection from rotavirus diarrhea are not well understood.

In the current study, we hypothesized that autophagy is activated during viral gastroenteritis and that LGG suppresses the virus-induced autophagy to prevent intestinal damage in infected pigs. We measured the effects of continuous LGG feeding in the gnotobiotic (Gn) pig model of virulent human rotavirus (HRV) gastroenteritis. Pigs were assigned to the following treatment groups: LGG plus HRV, HRV only, LGG only (LGG) or mock control. We investigated intestinal autophagy markers and pathologic changes in these groups. Here, we report that rotavirus infection alone, but not LGG alone, induced autophagy in the intestines of Gn pigs. LGG reduced the expression of autophagy markers to baseline levels and partially prevented tissue damage. Our study provides new insights into virus-induced autophagy and the role of LGG in suppressing autophagy and intestinal injury during viral gastroenteritis.

## Results

### Rotavirus infection, but not LGG treatment, induces autophagy in the Gn piglet intestine

**Figure 1:**
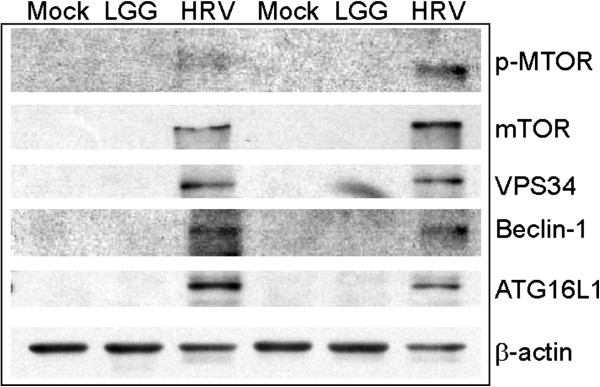
**Human rotavirus (HRV) stimulates autophagy proteins in gnotobiotic (Gn) pig ileum.** Autophagy proteins in pig ileum on post-HRV inoculation day (PID) 2. Tissue lysates were analyzed for p-mTOR (Ser 2448), total mTOR, VPS34, Beclin-1,and ATG16L1 by immunoblot. LGG, *Lactobacillus rhamnosus* GG.

We collected ileum tissues from the LGG mono-associated and/or rotavirus-infected Gn pigs on post-HRV inoculation day (PID) 2. We investigated the autophagy regulators mTOR and Class III PI3K VPS34 (also called PIK3C3) [15] and the autophagy markers ATG16L1 andBeclin-1. We found no significant changes in these markers in the pig intestine before or after LGG-monoassociation (Figure 1), indicating that LGG treatment alone had no effect on intestinal autophagy. In contrast, HRV infection elevated the protein levels of phospho-mTOR Ser 2448 (p-mTOR), mTOR, VPS34, Beclin-1, and ATG16L1 (Figure 1).

### LGG treatment protects the intestine from rotavirus-induced autophagy

**Figure 2:**
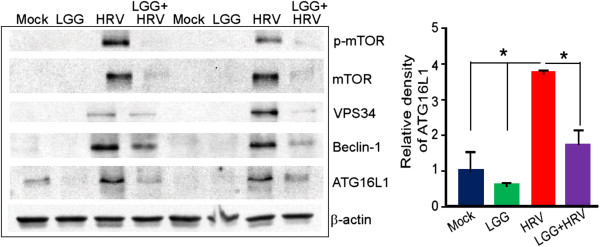
**LGG inhibits autophagy proteins enhanced by HRV in Gn pig ileum.** Autophagy proteins in pig ileum treated with LGG and HRV. Ileum epithelia were scraped on PID 2. Relative protein band intensities of ATG16L1 in pig ileum were analyzed with NIH image. Data are reported as the mean ± SD. ANOVA, * P<0.05.

To determine the effects of probiotic LGG on autophagy-related proteins, we immunoblotted for p-mTOR, mTOR, VPS34, ATG16L1, and Beclin-1 in small intestinal epithelial cells (Figure 2). LGG inhibited the expression of p-mTOR, mTOR, VPS34, Beclin-1 and ATG16L1, which were otherwise elevated by HRV infection (Figure 2).

### Location of autophagy markers in pig ileum

**Figure 3:**
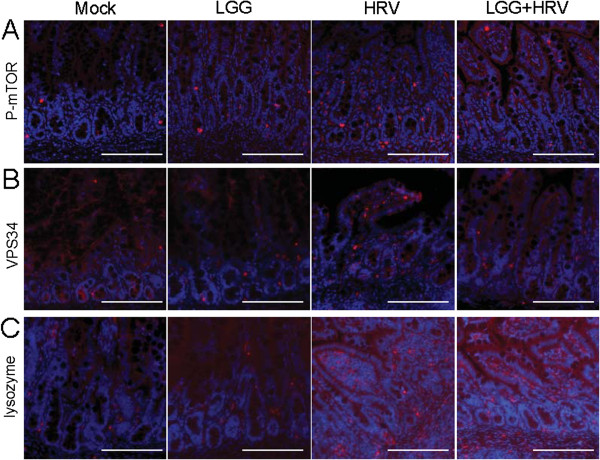
**Detection of p-mTOR, VPS34 and lysozyme proteins by immunofluorescence in pig ileum. (A)** HRV increases the expression of p-mTOR (Ser 2448) in pig ileum. Red, p-mTOR. **(B)** HRV increases the expression of VPS34 in Gn pig ileum. Red, VPS34. **(C)** HRV with/without LGG increases the expression of lysozyme in Gn pig ileum. Red, lysozyme; blue, DAPI (20× magnification, scale bar 100 μm).

We next investigated the location of autophagy markers in the pig ileum. In the mock control and LGG only groups, we noticed few p-mTOR-positive cells (Figure 3A). In the HRV-infected intestine, p-mTOR staining was more prevalent. LGG treatment decreased the number of p-mTOR-positive cells in HRV-infected intestine. VPS34 levels were similar to those of p-mTOR; LGG treatment suppressed the number of VPS34-positive cells (Figure 3B). Furthermore, immunostaining showed that lysozyme was increased in ilea from HRV and LGG+HRV pigs compared with mock and LGG only pigs (Figure 3C), which was consistent with the real-time PCR result that rotavirus infection induced high levels of lysozyme mRNA (data not shown).

### LGG treatment induces apoptosis in HRV-infected intestinal cells

**Figure 4:**
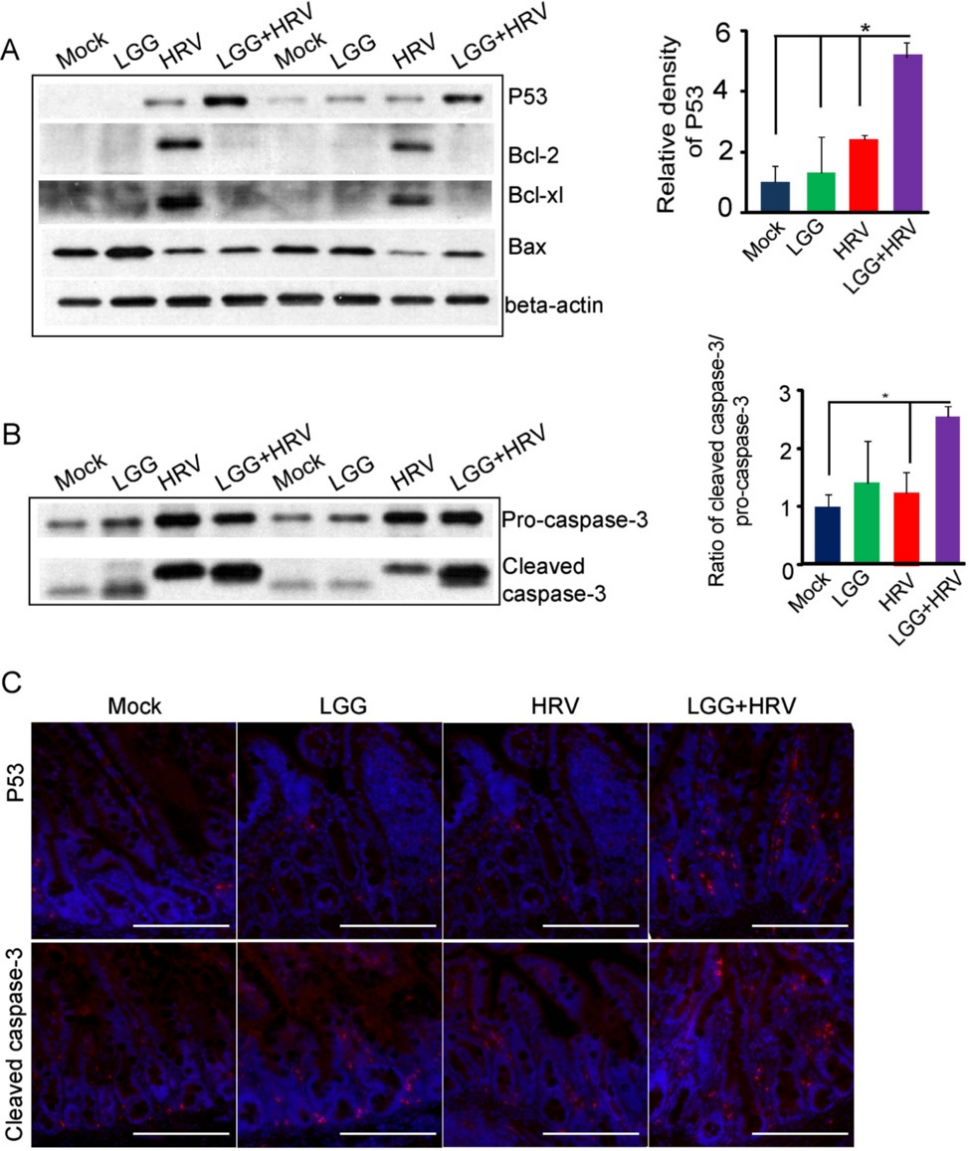
**LGG stimulates apoptosis in Gn pig ileum treated with HRV. (A)** LGG increases the expression of p53 protein and decreases Bcl-2 and Bcl-xl protein in the ilea of HRV-infected pigs. Ileum epithelia were scraped on PID 2. Relative protein band intensities of P53 in pig ileum were analyzed with NIH image. Data are reported as the mean ± SD. ANOVA * P<0.05. **(B)** LGG increases the expression of cleaved caspase-3 in the ilea of HRV-infected pigs. Densitometry analysis of caspase-3 activation. Values are the ratio of cleaved caspase-3 to pro-caspase-3. The results were normalized to mock (densitometer value = 1). * P<0.05. **(C)** Detection of p53 and cleaved caspase-3 by immunofluorescence in pig ileum (Magnification 20×, scale bar 100 μm).

Autophagy is a programmed survival strategy, whereas apoptosis is programmed cell death. We further examined the expression of apoptosis markers in the pig intestine (Figure 4A). p53 limits cellular proliferation by inducing cell cycle arrest and apoptosis in response to cellular stress [16,17]. LGG feeding with HRV infection led to higher p53 in the intestine of LGG+HRV pigs than in the other groups (Figure 4A). p53 staining showed an increased number of p53-positive cells in the ilea of the LGG+HRV group (Figure 4C). HRV infection also induced expression of the anti-apoptotic protein Bcl-2 and the pro-survival marker Bcl-xl, and these proteins were significantly decreased by LGG feeding (Figure 4A).

Bax is a pro-apoptotic Bcl-2-family protein [18] that resides in the cytosol and translocates to mitochondria upon induction of apoptosis [19]. While HRV infection suppressed Bax expression, LGG feeding inhibited this suppression of Bax, although there were no differences between the HRV and LGG+HRV pigs (Figure 4A). Bax also regulates caspase activity [20,21]. We measured cleaved caspase-3 to quantify cell apoptosis. The LGG+HRV pigs developed a higher ratio of cleaved caspase-3 to pro-caspase-3 (Figure 4B).

We then investigated the location of p53 in pig ileum. There were more p53-positive cells in the LGG+HRV pigs than in LGG and HRV only pigs (Figure 4C). Thus, LGG feeding increased the number of p53-positive intestinal cells in HRV-infected pigs. The localization of cleaved caspase-3 was similar to p53; LGG treatment enhanced the number of cleaved caspase-3-positive cells (Figure 4C).

### LGG feeding protects intestine physiology after virus infection

**Figure 5:**
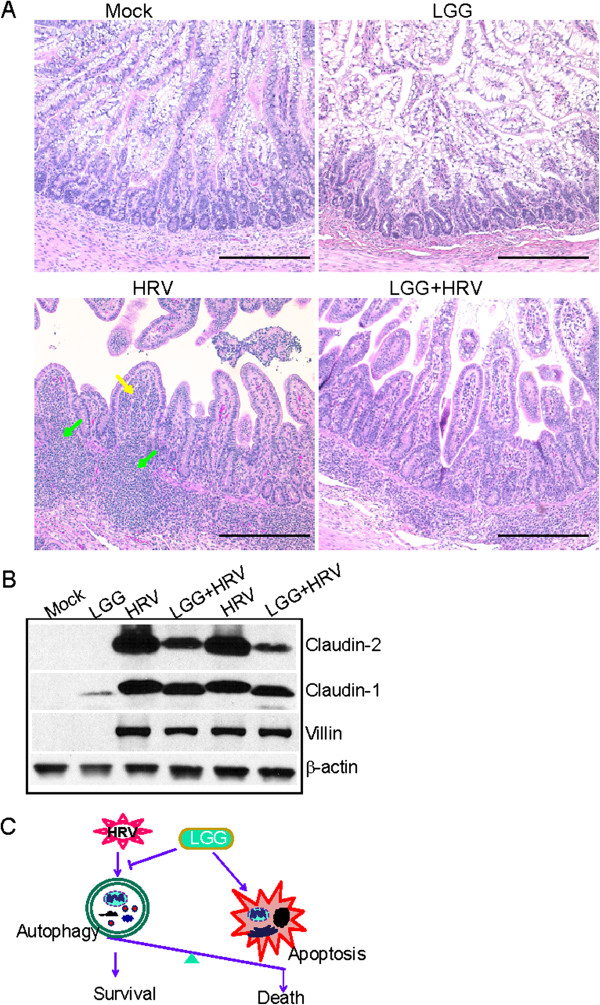
**LGG treatment protects intestine physiology after virus infection. (A)** H&E staining of the pig ileum. Infiltration of immune cells (yellow arrow) and discontinued muscularis mucosae (green arrow) were present in pig ilea infected with HRV (10× magnification, scale bar 200 μm). **(B)** HRV increases claudin-2 protein in pig ileum. **(C)** Diagram showing the potential roles of LGG in HRV-infected pig ileum. While HRV increases autophagy to establish infection in epithelial cells, LGG inhibits autophagy and increases apoptosis to clear HRV-infected cells.

LGG may suppress uncontrolled autophagy, thus protecting the intestine from injury during viral gastroenteritis. By H&E staining, we observed vigorous immune infiltration (yellow arrow) and discontinued muscularis mucosae (green arrow) in pig ileum infected with HRV (Figure 5A), but the LGG-treated infected ileum had less inflammation. LGG helped to maintain intestinal epithelial integrity. Claudin-2 is a ‘leaky’ claudin that forms a water channel, which mediates paracellular water transport in leaky epithelia [22-26]. Western blotting showed that virus infection increased the expression of claudin-2, whereas LGG treatment lowered the claudin-2 level after infection (Figure 5B). This result may explain our observations that LGG-treated piglets were less likely to develop HRV diarrhea and had a shorter mean duration of diarrhea compared with the non-treated group (data not shown). Our previous study and unpublished data showed that non-treated infected pigs had worse clinical and histopathological outcomes than the LGG+HRV group. Virus shedding was also lower in the LGG-treated pigs compared with the non-treated HRV group (data not shown).

## Discussion

In summary, our current studies made specific advances and uncovered several novel aspects of autophagy in rotavirus infection and probiotic protection: (1) rotavirus infection enhanced autophagy in the pig small intestine; (2) compared with mock treatment, oral LGG treatment in the Gn pig model did not change the expression of autophagy markers; and (3) LGG may suppress viral gastroenteritis by controlling the virus-induced autophagy responses in the intestine. Impaired autophagy is associated with the pathogenesis of various infectious diseases. Our data show that while rotavirus activated autophagy in the intestine at PID 2, LGG treatment down-regulated autophagy in response to viral infection, providing a balance between apoptosis and autophagy in the infected intestine (Figure 5C). In HRV-infected cells, LGG promotes apoptosis, which restricts virus spread to neighboring epithelia. LGG reduces rotavirus replication by suppressing the expression of autophagy proteins, which in turn impairs the assembly of infectious rotavirus particles [7]. Our study suggests a new molecular mechanism by which LGG suppresses uncontrolled autophagy and intestinal injury during viral gastroenteritis. Autophagy and apoptosis are connected both positively and negatively, and extensive crosstalk exists between them. Because our data showed that LGG-treated intestine had more apoptotic cells, LGG-induced apoptosis could help the host clear infected cells.

LGG treatment in infected Gn pigs reduced the expression of p-mTOR, total mTOR, VPS34, and Beclin-1 to normal levels in intestinal epithelial cells. These data suggest that LGG suppressed autophagy at the stage of initiation. Recent studies indicate that rotavirus and coronavirus hijack autophagy in the host [7,27]. Our data suggest that well-controlled autophagy is necessary to protect the host intestine from injury.

The Gn pig model allowed us to observe the effects of continuous LGG feeding on HRV gastroenteritis in a relatively clean background and well-controlled system. This model ensured that the data were not confounded by other microbes present in conventionally reared pigs. The gastrointestinal physiology and immune system in piglets closely mimic those of human infants [28]. However, we also recognize the limitations of the current studies. We had limited number of piglets in the experimental groups. We could not use a genetically engineered pig model to further investigate the host autophagy responses or the genes that mediate the protective role of LGG. In an *in vitro* experiment, we investigated autophagy after virus infection and LGG treatment in a pig intestinal epithelial cell line [29], but we did not observe responses similar to those *in vivo*. Moreover, some antibodies against the relevant porcine proteins are not commercially available. Regardless of the limitation, our current findings have their novelty in studying virus-induced autophagy and LGG suppression of uncontrolled autophagy *in vivo*.

In conclusion, we observed that LGG suppressed autophagy during rotaviral diarrhea. LGG treatment reduced rotavirus shedding and the incidence and duration of rotavirus diarrhea. We speculate that these probiotic effects are due to the regulation of autophagy in the intestine. Notably, we provide the first evidence of virus-induced autophagy in a well-controlled experimental gnotobiotic piglet model. LGG therapy has clear beneficial effects that protect the host from rotavirus diarrhea. Thus, a better understanding of the protective role of LGG during viral pathogenesis will lead to novel therapies for infectious diseases.

## Methods

### Ethics statement

The animal experiments in this study were approved (#10-168-CVM) by the Institutional Animal Care and Use Committee of The Virginia Polytechnic Institute and State University. The Virginia-Maryland Regional College of Veterinary Medicine (VMRCVM) is fully accredited by the American Association for Accreditation of Laboratory Animal Care (AAALAC) and approved by the Office of Laboratory Animal Welfare (OLAW) in compliance with the Public Health Service Policy on Humane Care and Use of Laboratory Animals. Euthanasia of pigs was conducted using a method consistent with the recommendations of the Panel on Euthanasia of the American Veterinary Medical Association.

### Rotavirus

The HRV Wa strain (G1P1A [14]) (from Dr. Linda Saif, The Ohio State University, USA) was passaged in Gn pigs, and the pooled intestinal contents of pigs were used for inoculation at a dose of ~10^5^ fluorescence forming units (FFU). The 50% infectious dose (ID_50_) of HRV in neonatal Gn pigs was determined to be approximately 1 FFU [30]. The virus titer was determined by cell culture immunofluorescence (CCIF) and was expressed as FFU/ml, as described previously [29].

### Probiotic bacteria strain

*Lactobacillus rhamnosus* GG (ATCC, 53103) cultures were anaerobically propagated (85% nitrogen, 10% hydrogen, 5% carbon dioxide) in Lactobacilli MRS broth (Weber Scientific, 3113–60) overnight at 37°C in sealed Gaspak jars containing Anaerogen packs. Bacterial suspensions (MRS with 15-20% glycerol) were stored at −80°C. Bacteria were titrated on MRS agar plates and quantified as CFU per ml, as described previously [31,32]. Prior to feeding, the bacteria were thawed, washed 2 times with 0.1% peptone water, centrifuged at 2000 rpm for 10 min at 4°C and diluted to specified CFU/ml.

### Gnotobiotic pig treatment

Near-term pigs were surgically removed from pregnant sows (Large White cross bred) and maintained in Gn isolator units as described [33]. All pigs were confirmed as germ-free prior to LGG feeding and sera negative for rotavirus antibodies prior to HRV exposure. Pigs (both males and females) were randomly assigned to four treatment groups as follows: (1) LGG colonization plus HRV infection (LGG+HRV), (2) HRV only (HRV), (3) LGG only (LGG) or (4) mock control (Mock). At 3 days of age (post-LGG feeding day, pigs in LGG groups were orally dosed with 10^3^ CFU LGG in 3 ml of 0.1% peptone water, and the daily LGG dose increased by 10-fold each day up to 10^12^ CFU. The incremental increases in LGG doses were determined empirically to avoid causing diarrhea by high doses of probiotic bacteria during the first few days of life, as Gn pigs lack maternal antibodies. The pigs were then fed 10^12^ CFU every day until euthanasia. Pigs in non-LGG groups were given an equal volume of 0.1% peptone water. At PFD 9 (post-HRV inoculation day [PID] 0), pigs in HRV groups were orally inoculated with10^5^ FFU HRV in 5 ml Diluent #5 (Modified Eagle Medium with 1% non-essential amino acids, 1% antibiotics [penicillin 10,000 I.U./ml + streptomycin 10,000 MCG/ml + amphotericin 25 MCG/ml]), and pigs in non-HRV groups were orally inoculated with an equal volume of Diluent #5. Pigs were given 8 ml of 100 mM sodium bicarbonate 20 min before HRV inoculation to reduce gastric acidity. Pigs were euthanized for sample collection on PFD11 (PID 2).

### Histology and immunofluorescence staining

Distal ileal tissues were freshly isolated and paraffin-embedded after fixation with 10% neutral buffered formalin. For histology, the slides were processed with Hematoxylin-Eosin (H&E) staining and observed under a microscope. Immunofluorescence was performed on paraffin-embedded sections (5 μm). After preparation of the slides as described previously [34], tissue samples were incubated with anti-lysozyme (Santa Cruz, sc27958), anti-p-mTOR (Ser 2448) (Cell Signaling, 5536), anti-VPS34 (Invitrogen, 382100), anti-p53 (Santa Cruz, sc-126), or anti-cleaved caspase-3 (Biocare Medical, CP229C). Samples were then incubated with Donkey anti-goat Alexa Fluor 594 (Invitrogen, A11058), goat anti-rabbit Alexa Fluor 594 (Invitrogen, A11037) or goat anti-mouse Alexa Fluor 594 (Invitrogen, A11032) and DAPI (Invitrogen, D1306) for 1 hour at room temperature. Tissues were mounted with SlowFade (Invitrogen, S2828) under a cover slip, and the edges were sealed to prevent drying. Specimens were examined with a Leica SP5 Laser confocal microscope.

### Western blot analysis and antibodies

Autophagy and apoptosis-related proteins were detected by western blot of pig ileum sections and ileal epithelial cells. The ileal epithelial cells were collected by cutting or scraping, as previously described [35]. Briefly, epithelial cells were lysed in a lysis buffer (1% Triton X-100, 150 mmol/L NaCl, 10 mmol/L Tris pH 7.4, 1 mmol/L EDTA, 1 mmol/L EGTA pH 8.0, 0.2 mmol/L sodium ortho-vanadate) containing a protease inhibitor cocktail (Roche Diagnostics, 11836170001). Equal amounts of protein were separated by SDS-polyacrylamide gel electrophoresis, transferred to nitrocellulose (Bio-Rad, 162–0112), and immunoblotted with the following primary antibodies: anti-phospho-mTOR (ser2448) (Cell Signaling, 5536), anti-mTOR (Cell Signaling, 2983), anti-caspase-3 (Cell Signaling, 9665), anti-VPS34 (Invitrogen, 382100), anti-Beclin-1 (Santa Cruz, sc-10086) , anti-P53 (Santa Cruz, sc-126), anti-Bcl-2 (Santa Cruz, sc-7382), anti-Bcl-xl (Santa Cruz ,sc-8392), anti-Villin (Santa Cruz, sc-7672), anti-ATG16l1 (Abgent, AP18176), or anti-β-actin (Sigma-Aldrich, A5441). Proteins were visualized by ECL chemiluminescence. Protein bands of interest were quantitated densitometrically and normalized to β-actin.

## Competing interests

The authors declare that they have no competing interests.

## Authors’ contributions

SW, YZ, FL, GL, KW, JK, XY: performing experiments, contribution to concept and design, analysis and interpretation of data, and important intellectual input in drafting or revising the manuscript. LY, JS: contribution to concept and design, analysis and interpretation of data, and important intellectual input in drafting or revising the manuscript.
